# Nasal Continuous Positive Airway Pressure as a Preferred Airway Management During IV Sedation of Obese Patients With Obstructive Sleep Apnea Undergoing Functional Luminal Imaging Probe Panometry

**DOI:** 10.7759/cureus.28509

**Published:** 2022-08-28

**Authors:** Mahdi Abdallah, Anh Nguyen, Nimit Kasliwal, Daniel Gunn, Sergio Murillo, Saravanan Ramamoorthy

**Affiliations:** 1 Department of Anesthesiology, Texas A&M College of Medicine, Dallas, USA; 2 Department of Gastrointestinal Surgery, Baylor University Medical Center, Dallas, USA; 3 Department of Anesthesiology, Baylor University Medical Center, Dallas, USA

**Keywords:** functional luminal imaging probe, cpap, continuous positive airway pressure, obstructive sleep apnea, airway management, endoscopic management of obesity, difficult airway management, obstructive sleep apnea (osa), continuous positive airway pressure (cpap)

## Abstract

The functional luminal imaging probe (FLIP) utilizes high-resolution planimetry to provide information regarding esophagogastric junction (EGJ) diameter, EGJ distensibility, and reactive contractile patterns of the esophageal body. This is an FDA-approved measurement tool utilized to both diagnose and measure various upper gastrointestinal disorders. While patients are sedated during FLIP panometry, significant respiratory variations can affect the quality of FLIP panometry results. Nasal continuous positive airway pressure (CPAP) can be utilized to prevent intraoperative or postoperative hypoxia in obese patients as well as those with obstructive sleep apnea (OSA). In this retrospective chart review, we compared obese patients with a diagnosis of OSA who underwent FLIP panometry utilizing nasal CPAP as airway management against a group who underwent the same procedure with a nasal cannula to evaluate the incidence of hypoxia, hypercapnia, variation in cardiovascular dynamics, and the quality of FLIP panometry readings.

## Introduction

Functional luminal imaging probe (FLIP) panometry is a novel tool to further evaluate the esophageal function as well as guide management and treatment in patients with dysphagia or other esophageal symptoms [1,2]. Placed during upper endoscopy, the FLIP catheter contains impedance sensors encased in a compliant water-filled balloon with a distal pressor sensor allowing for measurement of esophageal surface area and pressure at different balloon volumes. Quality of sedation, intraoperative movement, coughing, and repetitive retrograde contractions (RRCs) can affect the quality of FLIP panometry results. The presence of RRCs at low balloon volumes on FLIP panometry is thought to be due to respiratory variations introduced during a surgical procedure. Obese patients or those with a diagnosis of obstructive sleep apnea (OSA) are particularly at risk for hypoxia during anesthesia induction [3]. Nasal continuous positive airway pressure (CPAP) with a CPAP machine and mask has been shown to be effective at minimizing hypoxia in obese and OSA patients undergoing sedation [4-6]. Our retrospective chart review study aimed to evaluate whether the use of nasal CPAP as airway management in obese patients with OSA undergoing FLIP panometry would minimize hypercapnia, hypoxia, and cardiovascular variations and help improve FLIP panometry quality readings.

## Materials and methods

In this retrospective chart review, 50 obese patients with an apnea-hypopnea index (AHI) > 15, who were candidates for gastric bypass surgery at Baylor University Medical Center (BUMC) with a chart diagnosis of OSA, who underwent upper endoscopy with FLIP panometry from April to July 2021, were included. This retrospective chart review analysis was IRB Quality Improvement approved by our institution. Based on provider comfort and experience, some patients received nasal CPAP as preferred airway management and others received nasal cannula instead. Group 1 (25 patients) had the nasal CPAP utilized for airway management, while group 2 (25 patients) had the nasal cannula utilized for airway management during FLIP panometry. In this study, group 2 was selected as the control group due to the use of a nasal cannula as the standard method of airway management and sedation. The nasal CPAP settings were standard set for these patients at pressure support of 10 cmH2O and oxygen flow of 10 L/min. The nasal cannula settings were also standard set with an oxygen flow of 4 L/min. All patients had a Patient State Index (PSI) value of 25-50 from SEDline (SEDLine, Inc., Irvine, CA) with optimal hypnotic sedation using propofol. The baseline past medical history for each group is displayed in Table 1 below. A single episode of hypoxia was defined as saturated oxygen concentration (SaO2) < 95% sustained for a duration of > two minutes. A single episode of hypercapnia was defined as end-tidal carbon dioxide > 40. An episode of elevated blood pressure (BP) was defined as systolic blood pressure (SBP) > 140 mmHg or diastolic blood pressure (DBP) > 100 mmHg sustained for a duration of > two minutes. Similarly, an episode of elevated heart rate (HR) was defined as HR > 90 beats per min (BPM) sustained for > two minutes. RRCs were extracted from the FLIP panometry data. These particular values were chosen as they are considered major changes in intraoperative vitals assuming each patient group was under the same amount of sedation. The average age was 56.8 years with 64% being female and an average BMI of 35.3. There was a high incidence of hypertension (64%) and gastroesophageal reflux disease (78%) in the study population. RRCs were identified from the FLIP panometry.

**Table 1:** Group 1 vs. group 2 patient baseline information CPAP: continuous positive airway pressure.

Airway management	Sample size	Average age	History of hypertension	History of gastroesophageal reflux disease	History of diabetes mellitus
Group 1 - nasal CPAP	25	60	72%	92%	28%
Group 2 - nasal cannula	25	57	56%	64%	28%

## Results

Table 2 summarizes the total number of intraoperative episodes of hypoxia, hypercapnia, elevated BP, and elevated HR for each group. Also conveyed in Table 2 are the total number of FLIP procedural interruptions and the percentage of RRCs produced by each group. One of the most significant differences between both groups was the total number of episodes of hypoxia. There was only one episode of hypoxia in group 1 while group 2 had a total of 32 episodes of hypoxia during the procedure. The total number of episodes of elevated BP was higher in group 2 (29 episodes) compared to group 1 (19 episodes). Similarly, the total number of episodes of elevated HR was higher in group 2 (27 episodes) compared with group 1 (10 episodes). The total number of episodes of hypercapnia was higher in group 2 (18 episodes) compared to group 1 (0 episodes). The total number of FLIP procedural measurement interruptions that occurred during the procedure was higher in group 2 (22 interruptions) compared to group 1 (three interruptions). Finally, Table 2 shows the FLIP panometry results with a mean esophagogastric junction (EGJ) diameter of 12.1 mm and EGJ distensibility of 3.9 mm2/mmHg at the 60 mL balloon volume. Secondary reactive contractile patterns at the 60 mL balloon volume in this obese cohort with OSA included absent contractile response (52%), borderline contractile response (4%), impaired/disordered contractile response (24%), and spastic reactive contractile response (8%). The presence of RRCs at 30-60 mL balloon volume was higher in group 2 (36%) compared to group 1 (16%).

**Table 2:** Group 1 vs. group 2 intraoperative variables comparison (cumulative episodes) CPAP: continuous positive airway pressure; FLIP: functional luminal imaging probe; BP: blood pressure; HR: heart rate.

Airway management	Sample size	Hypoxia (episodes)	Elevated BP (episodes)	Elevated HR (episodes)	Hypercapnia (episodes)	FLIP procedural interruptions (episodes)	Repetitive retrograde contractions
Group 1 - nasal CPAP	25	1	19	10	0	3	16%
Group 2 - nasal cannula	25	32	29	27	18	22	36%

**Figure 1:**
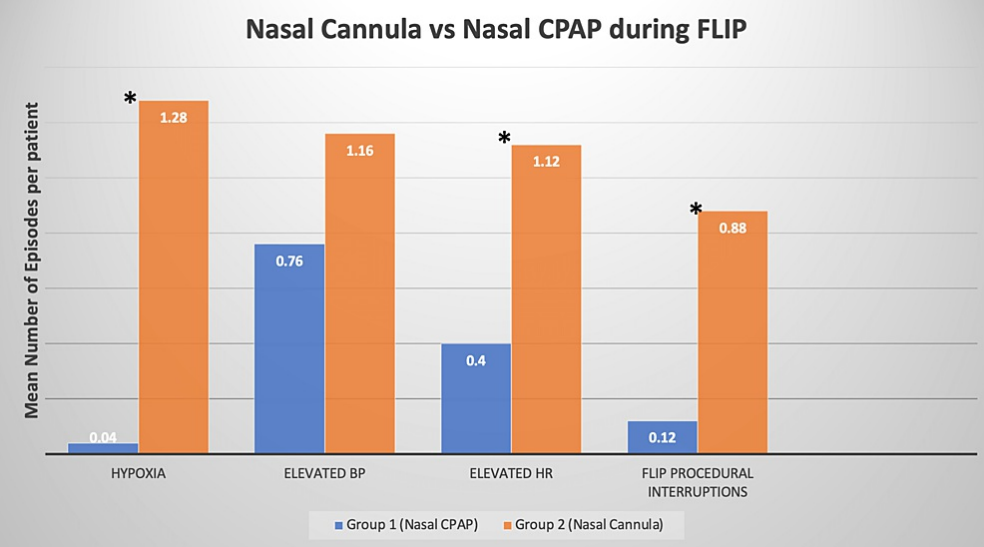
Group 1 vs. group 2: mean episodes of each intraoperative vital sign per patient in each group CPAP: continuous positive airway pressure; FLIP: functional luminal imaging probe; BP: blood pressure; HR: heart rate.

Figure 1 below displays the average number of episodes of hypoxia, elevated BP, elevated HR, and FLIP procedural interruptions for each patient in each group. For instance, we can see that the average number of episodes of hypoxia per patient in group 1 was 0.04 whereas group 2 had an average of 1.28 hypoxic episodes per patient. A two-tailed independent t-test analysis was undergone on each of the variables presented in Figure 1. The statistical analysis delineated that there was a significant difference in the average number of episodes of hypoxia (p = <0.001), elevated HR (p = 0.018), and FLIP procedural interruptions (p = 0.00016). However, it failed to show a significant difference in the average number of episodes of elevated BP (p = 0.13) between each group. Moreover, an N-1 chi-square test showed a significant decrease in the percentage of RRCs (p < 0.05) in group 1 (16%) when compared to group 2 (36%).

## Discussion

This retrospective chart review highlights the potential benefit of utilizing nasal CPAP for airway management in obese patients with OSA undergoing FLIP panometry under IV sedation. Aside from their use intraoperatively for airway management, CPAP machines have been a well-established treatment for patients outside of surgery with OSA as they have been shown to decrease overall blood pressure, daytime sleepiness, risk of heart failure, and stroke [7]. Besides its ability to significantly reduce intraoperative hypoventilation, hypercapnia, and hypoxia, the use of CPAP as airway management decreased episodes of RRCs at low balloon volumes, which may represent respiratory variations on FLIP panometry. RRCs are hypothesized to be indicative of either impaired inhibitory innervation, esophageal outflow obstruction, or sustained esophageal distension [8]. RRCs appear to be a manifestation of spastic esophageal dysmotility with an increased risk of aspiration. Patients with OSA may potentially have several episodes of RRCs because OSA leads to the development of many esophageal functional changes [9]. Therefore, these functional changes instigate the impairment of esophageal inhibitory innervation and outflow obstruction that is conveyed on the FLIP panometry as RRCs. The pathophysiology of this may be due to decreased episodes of intraoperative pharyngeal airway obstruction with CPAP. Recurrent obstructions lead to disruptions in physiological breathing, thereby causing hypercapnia and hypoxia as well as surges of sympathetic activation [7]. The hypercapnic-induced sympathetic activation further increases the intraoperative movement of the patient, which can affect the quality of FLIP panometry results and the increase in the number of RRCs. This would also explain the significant discrepancy in the number of FLIP procedural interruptions between both groups. The patient’s intraoperative movement during the procedure likely induced by sympathetic activation secondary to hypercapnia and hypoxia is interrupting the reading of FLIP panometry. In addition, the use of nasal CPAP can decrease the incidence of RRCs by increasing the intrapleural pressure, which would in turn increase the duration of constriction of the lower esophageal sphincter [10]. As the incidence of RRCs is significantly decreased in the nasal CPAP group, the risk of aspiration could be potentially decreased as well [11]. Therefore, the use of nasal CPAP as preferred airway management for patients with OSA can provide a consistent supply of oxygen to keep the patient’s airway open throughout the FLIP panometry procedure and thereby prevent any pharyngeal airway obstruction that otherwise leads to intraoperative movements secondary to hypercapnia-induced sympathetic activation translating into the decreased quality of the FLIP panometry results.

## Conclusions

In summary, in this retrospective chart review, we compared obese patients with a diagnosis of OSA who underwent FLIP panometry utilizing nasal CPAP as preferred airway management against a group who underwent the same procedure utilizing the standard nasal cannula as airway management to evaluate the incidence of hypoxia, hypercapnia, variation in cardiovascular dynamics, and the quality of FLIP panometry readings. This study has potential limitations. With a larger sample size, a more accurate statistical analysis could be attempted to compare the intraoperative vitals of the study groups. In addition, the nature of the study type produced many limitations owing to the design of a retrospective chart review as there could have been potential biases in our data and results. Being a retrospective chart review, the possibility of a selection bias is present due to convenience sampling and thus each group may not accurately represent the general population. We understand that to further evaluate the superiority of nasal CPAP as preferred airway management during FLIP procedures in patients with OSA, we must further undergo a randomized prospective study analysis. The nasal CPAP’s ability to provide a steady flow of oxygen or steady maintenance of pressure keeps the patient's airways open continuously throughout the FLIP panometry procedure, thus preventing intraoperative hypoxia and hypercapnia as well as minimizing respiratory variations, which may affect FLIP panometry findings. CPAP is able to increase intrapleural pressure thereby preventing constriction of the lower esophageal sphincter to decrease RRCs. Moreover, as the incidence of RRCs is significantly decreased in the nasal CPAP group, the risk of aspiration is hypothesized to follow and decrease as well. Therefore, by minimizing episodes of intraoperative hypoxia and hypercapnia, we can blunt any intraoperative movements caused by hypercapnic-induced sympathetic activation and thereby improve the quality of the FLIP panometry readings.
